# Treatment patterns and outcomes in BRAF V600E-mutant melanoma patients with brain metastases receiving vemurafenib in the real-world setting

**DOI:** 10.1002/cam4.475

**Published:** 2015-05-20

**Authors:** Geoffrey T Gibney, Geneviève Gauthier, Charles Ayas, Philip Galebach, Eric Q Wu, Sarang Abhyankar, Carolina Reyes, Annie Guérin, Yeun Mi Yim

**Affiliations:** 1 Moffitt Cancer CenterTampa, Florida; 2 Analysis Group Inc.Boston, Massachusetts; 3 Genentech Inc.South San Francisco, California

**Keywords:** BRAF mutation, brain metastases, melanoma brain metastases, metastatic melanoma, vemurafenib

## Abstract

Brain metastases are a common and serious complication among patients with metastatic melanoma. The selective BRAF inhibitor vemurafenib has demonstrated clinical efficacy in patients with BRAF V600E-mutant melanoma brain metastases (MBM). We examined the real-world application and clinical outcomes of vemurafenib in this patient population. Demographic, treatment patterns, response, and survival data were collected from medical charts. Clinical data on 283 patients with active BRAF V600E-mutant MBM treated with vemurafenib were provided by 70 US oncologists. Mean age was 57.2 years, 60.8% were male, 67.5% had ECOG performance status of 0–1, and 43.1% used corticosteroids at vemurafenib initiation. Median follow-up was 5.7 months. Following vemurafenib initiation, 48.1% of patients experienced intracranial response and 45.6% experienced extracranial response. The Kaplan–Meier estimate for overall survival was 59% at 12 months. Multivariate analyses showed associations between intracranial response and both corticosteroid use and vemurafenib as initial therapy after MBM diagnosis. Larger size (5–10 mm vs. <5 mm) and number of brain metastases (≥5 vs. <2) and progressive extracranial disease at treatment initiation were associated with decreased intracranial response and increased risk of disease progression. Multiple extracranial sites (2 vs. <2) and the absence of local treatments were also associated with increased risk of progression. Increased risk of death was associated with ≥2 extracranial disease sites, progressive extracranial disease, and ≥5 brain metastases. Subgroups of MBM patients may derive more benefit with vemurafenib, warranting prospective investigation.

## Introduction

Among patients with metastatic melanoma, brain metastases are a common and serious event, contributing to 20–50% of melanoma-related deaths 1. In newly diagnosed-stage IV patients, brain metastases are present in approximately 20% of cases 2, and up to 75% of patients develop brain metastases over the course of the disease 3. The prognosis for these patients is typically poor. Median overall survival (OS) is estimated to be 3–5 months 4–6. Aggressive approaches such as surgery and stereotactic radiosurgery (SRS) may extend survival to over 8 months; however, this is limited to the subset of patients who are candidates for such procedures 7. Whole-brain radiation therapy (WBRT) is typically reserved for patients with a larger number of brain metastases or after intracranial failure of surgery or SRS, but survival is only marginally improved 5–8.

In addition to surgery and radiation therapies, systemic treatments such as chemotherapy and immunotherapy have been investigated for the management of melanoma brain metastases (MBM). Clinical trial results of MBM patients treated with temozolomide and fotemustine alone or in combination with thalidomide or radiation have shown response rates of only 7–12% 9–13. For high-dose interleukin-2, a retrospective study reported a response rate of 5.6% for intracranial and extracranial sites in previously untreated MBM patients 14. In the phase II study of ipilimumab in patients with active MBM, objective intracranial response rates were 16% in asymptomatic patients not receiving corticosteroids at study entry and 5% in patients with symptomatic brain metastases 15. Higher response rates have been reported in retrospective studies of adoptive cell therapy in patients with active MBM, but its use has been limited by its complexity and potential toxicities 16.

Approximately 50% of metastatic melanomas contain an activating BRAF mutation, which is most often located on codon 600 (BRAF V600 mutation, part of the mitogen-activated protein kinase [MAPK] pathway) 17, 18. Mutant BRAF kinase can be inhibited by the use of selective BRAF inhibitors (such as vemurafenib and dabrafenib), thereby blocking the constitutive activation of the MAPK pathway 19–21. Prospective studies of dabrafenib and vemurafenib in patients with BRAF V600-mutant MBM have shown promising results. In the phase II study of dabrafenib, investigator-assessed intracranial response was observed in 39.2% of patients without prior local treatment and 30.8% of patients with prior local treatment (BRAF V600E mutant); median progression-free survival (PFS) was 3.8 and 3.9 months, respectively 17. In the initial pilot study of vemurafenib in patients with active BRAF V600E MBM, objective intracranial response was observed in 16% (3/19) patients and median PFS was 3.9 months 22. Vemurafenib was further investigated in an open label phase II study with BRAF V600E-mutant MBM patients (Cobas® positive) 18. Investigator-assessed intracranial response was observed in 29% of patients without prior local treatment and 21% of patients with prior local treatment; median PFS was 3.7 and 4.0 months, respectively 23. The most common adverse reactions to vemurafenib include joint pain, rash, hair loss, fatigue, photosensitivity reactions, nausea, pruritus, and skin papilloma 24. Adverse events reported rarely required permanent vemurafenib discontinuation and were generally manageable with dose reduction or interruption 25. Incidence of grade 4 adverse events occurred in ≤4% of cases 24.

Despite promising results from phase II studies of BRAF inhibitors in MBM patients, information regarding treatment outcomes in a “real-world” setting remains limited. The primary objectives of this retrospective study were to determine treatment response and OS in patients with active BRAF V600E-mutant MBM treated with vemurafenib. As secondary objectives, this study aimed to identify clinical variables associated with intracranial treatment response, disease progression, and mortality.

## Methods

### Data collection

Patient chart information was collected via an online data extraction form completed by a panel of US medical oncologists. Participating oncologists provided information on up to ten BRAF V600E MBM patients treated with vemurafenib on or after August 17, 2011. Eligible patients were required to be ≥18 years at vemurafenib initiation, confirmed to have BRAF V600E-mutant metastatic melanoma and active brain metastases (defined as nonradiated and nonresected brain metastases appearing on imaging studies or previously radiated or resected brain metastases with evidence of progression). Patients were excluded if any BRAF inhibitor was administered in a clinical trial, complete medical records were not available, or serial radiographic assessment of brain metastases were unavailable to the responding physician.

Clinical data abstracted from medical charts included demographics, clinical characteristics, treatment history, vemurafenib treatment characteristics and outcomes, and vital status. Due to retrospective data collection in a nontrial setting, the following response definitions were provided to ensure comparability in the assessment of tumor response: (1) complete response (CR): disappearance of all lesions, (2) partial response (PR): decreased size in the majority of lesions with no new lesions, (3) progressive disease (PD): enlargement of existing lesions, appearance of new lesions, or other clinical evidence of PD, and (4) stable disease (SD): absence of CR, PR, and PD. IRB exemption was obtained for this study, as all data were de-identified.

### Statistical methods

The vemurafenib initiation date was defined as the study index date. Demographic, clinical, and treatment characteristics were descriptively reported with their associated percentage and interquartile range (IQR). Overall intracranial and extracranial treatment responses were defined as the achievement of CR or PR while treated with vemurafenib. Kaplan–Meier (KM) analyses were used to estimate OS rates at 6 and 12 months from the time of vemurafenib initiation. Patients were censored at the date of last follow-up if still alive. OS rates were reported for the overall sample and stratified by prior local treatment for brain metastases. Due to limited follow-up, physicians were contacted 6 months after the original data collection to obtain updated vital status; KM analyses were used to estimate the OS rates using the updated information. Sensitivity analyses were conducted by excluding patients with short follow-up duration from the KM analyses.

Logistic regression models were used to identify factors associated with intracranial treatment response and intracranial disease progression. Cox proportional-hazard regression models were used to identify factors associated with increased risk of mortality. Clinically relevant variables were selected based on previous publications on MBM. Using a stepwise approach, variables that did not significantly contribute to the likelihood of achieving treatment response, to the risk of intracranial disease progression, or to the risk of death were excluded until the final model was obtained. Results for the logistic and Cox models were reported as odds ratios and hazard ratios, respectively, with their 95% confidence intervals and *P*-values using a two-sided test with a 5% significance level.

## Results

A total of 70 oncologists participated in the study and provided data on 283 eligible patients. The majority of oncologists had more than 5 years of practice (91.4%) and practiced in a community setting (71.4%).

### Patient demographic, clinical, and treatment characteristics

The mean age of patients at vemurafenib initiation was 57 years, 60.8% were male, and 62.9% were non-Hispanic White (Table1). Hypertension (36.0%) and diabetes (27.2%) were the most commonly reported comorbidities. Most patients had Eastern Cooperative Oncology Group (ECOG) performance status of 0–1 and 34.3% patients had an elevated LDH level. The median number of brain metastases was 2 (IQR 1.0; 3.0) and the median diameter for the largest brain lesion was 10 mm (IQR 5.0; 18.0). Approximately half of the patients had ≥2 extracranial metastatic sites (50.2%). Prior local treatment for brain metastases (surgery and/or radiation therapy) was reported for 38.5% of the patients.

**Table 1:** Patient characteristics^1^

	*N* = 283
Age, years, mean ± SD	57.2 ± 11.5
Male, *n* (%)	172 (60.8)
Race or ethnicity, *n* (%)	
Non-Hispanic White	178 (62.9)
Hispanic or Latino	61 (21.6)
Black	24 (8.5)
Asian or Pacific Islander	13 (4.6)
Unknown	7 (2.5)
Comorbidities, *n* (%)^2^	
Hypertension	102 (36.0)
Diabetes mellitus (type I or II)	77 (27.2)
Digestive disorders	38 (13.4)
Chronic obstructive pulmonary disease (COPD)	33 (11.7)
Cardiovascular diseases	31 (11.0)
ECOG performance status, *n* (%)	
0–1	191 (67.5)
≥2	85 (30.0)
Unknown	7 (2.5)
Site of primary melanoma, *n* (%)	
Cutaneous	227 (80.2)
Acral	34 (12.0)
Mucosal	33 (11.7)
Other	4 (1.4)
Elevated LDH, *n* (%)	97 (34.3)
Median diameter of largest brain metastases, mm (IQR)	10.0 (5.0, 18.0)
Number of brain metastases, median (IQR)	2.0 (1.0–3.0)
Number of extracranial metastatic sites, *n* (%)	
0	43 (15.2)
1	98 (34.6)
2–4	141 (49.8)
>4	1 (0.4)
Prior systemic therapy, *n* (%)	125 (44.2)
Prior local treatment for brain metastases, *n* (%)	109 (38.5)
Stereotactic radiosurgery	48 (17.0)
Whole-brain radiosurgery	34 (12.0)
Surgery	30 (10.6)

SD, standard deviation; ECOG, Eastern Cooperative Oncology Group; LDH, lactate dehydrogenase; IQR, interquartile range.

1 Patient characteristics are summarized at the time of vemurafenib initiation or the latest examination prior to vemurafenib initiation.

2 Comorbidities with a prevalence of ≥10% reported.

Median follow-up time after vemurafenib initiation was 5.7 months (Table2). Concomitant use of vemurafenib and corticosteroids was observed in 43.1% of patients. For the majority of patients (75.0%), the dose of corticosteroids was lowered or discontinued over the course of vemurafenib therapy. At the time of data collection, 37.5% of patients continued to receive vemurafenib, 9.5% of patients had died while on vemurafenib, and 53.0% of patients had discontinued vemurafenib. The most common reasons for discontinuation were systemic disease progression (50.7%) and intracranial disease progression (21.3%) (Table2). The reason for discontinuation was unknown in 34.7% of patients that stopped vemurafenib treatment. Among patients who discontinued vemurafenib, the median duration of treatment was 4.8 months (IQR 2.8; 7.7).

**Table 2:** Vemurafenib treatment characteristics

	*N* = 283
Time from vem initiation to last follow-up visit, median (IQR), months	5.7 (3.0, 9.2)
Line of therapy at treatment initiation, *n* (%)^1^	
First line	260 (91.9)
Later lines	23 (8.1)
Concurrent corticosteroids treatment, *n* (%)	122 (43.1)
Use of corticosteroids at the time of vem initiation, *n* (%)	76 (26.9)
Maintained total daily dose	13 (17.1)
Increased total daily dose	6 (7.9)
Lowered total daily dose or tapered corticosteroid use	57 (75.0)
Vem treatment discontinuation, *n* (%)	150 (53.0)
Reason for vem treatment discontinuation, *n* (%)^2^	
Systemic disease progression	76 (50.7)
Intracranial disease progression	32 (21.3)
Drug toxicity/drug intolerance	1 (0.7)
Other	13 (8.7)
Unknown	52 (34.7)

IQR, interquartile range; Vem, vemurafenib.

1 Use of vemurafenib after the diagnosis of brain metastases.

2 Respondents were allowed to provide more than one reason for vem discontinuation. Categories are not mutually exclusive.

### Treatment response and survival

While on vemurafenib treatment, 48.1% of patients achieved an intracranial response and 45.6% achieved an extracranial response (Table3). Stable intracranial and extracranial disease was reported in 19.1% and 20.1% of patients, respectively. Overall, 27.6% and 35.7% of patients experienced intracranial and extracranial disease progression while on vemurafenib, respectively.

**Table 3:** Treatment response to vemurafenib

	All patients (*N* = 283)	
	Intracranial	Extracranial
Best response, *n* (%)		
Complete or partial response	136 (48.1)	129 (45.6)
CR: disappearance of all lesions	40 (14.1)	32 (11.3)
PR: decreased size in majority of existing lesions with no new lesions	96 (33.9)	97 (34.3)
Progressive disease	49 (17.3)	53 (18.7)
Enlargement of existing lesions	35 (12.4)	39 (13.8)
Appearance of new lesions	19 (6.7)	26 (9.2)
Other clinical evidence of progressive disease	1 (0.4)	0 (0.0)
Stable disease: none of the above	54 (19.1)	57 (20.1)
Unknown	44 (15.5)	44 (15.5)

CR, complete response; PR, partial response.

**Figure 1:**
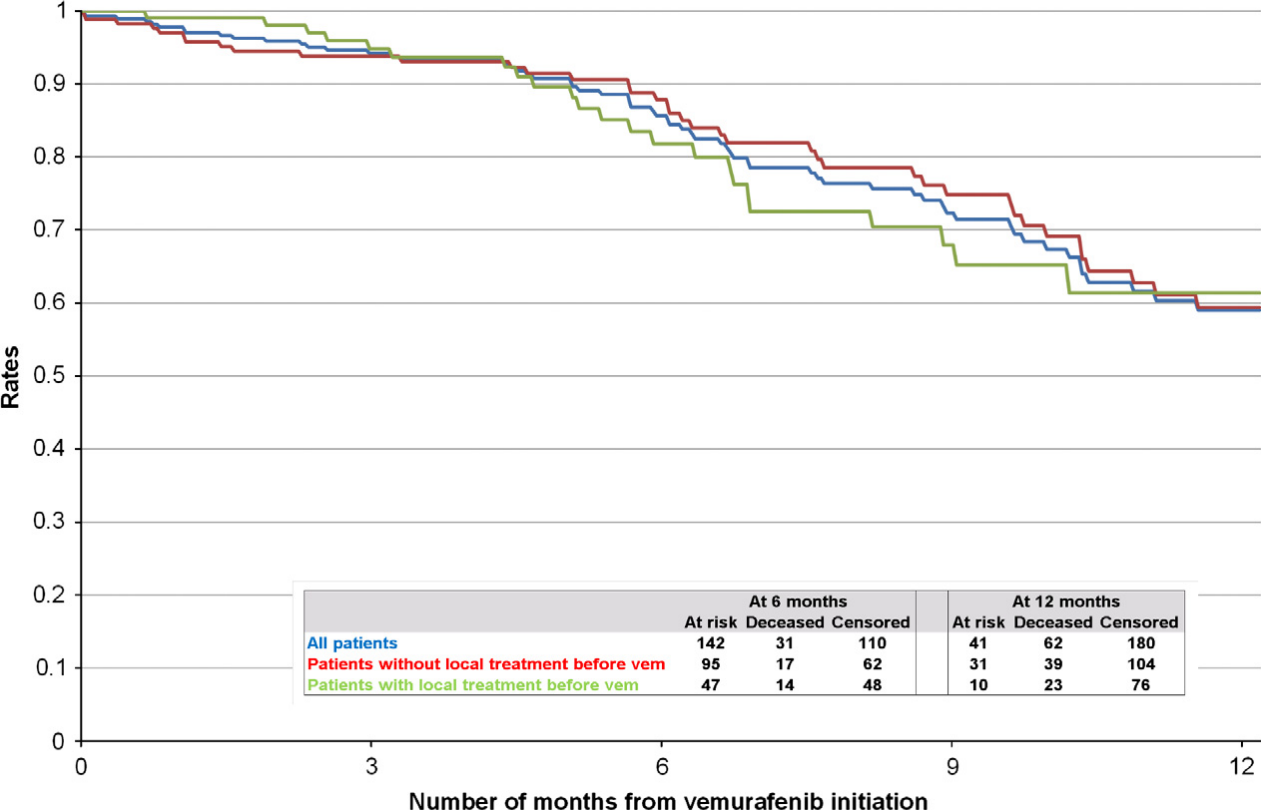
Overall survival rates.

At the time of the initial data collection, 70 patients (24.7%) were deceased. No deaths were indicated as drug related. The overall KM estimates of OS rates were 85.6% (CI; 80.1; 89.8) at 6 months and 59.0% (CI; 49.5; 67.2) at 12 months (Fig.1). For patients with and without prior local treatments, rates were 81.8% (CI; 70.9; 88.9) and 87.9% (CI; 81.0; 92.3) for the 6-month period and 61.4% (CI; 45.9; 73.6) and 59.4% (CI; 47.7; 69.3) for the 12-month period, respectively. Physicians were contacted 6 months after the original data collection to update the vital status of patients initially reported as alive. In updated analyses, 98 patients (34.6%) were deceased and median follow-up time increased to 7.0 months. Updated OS and sensitivity analyses (the latter excluded patients with short follow-up) yielded similar results (Appendix 1).

### Predictors of patient outcomes

**Figure 2:**
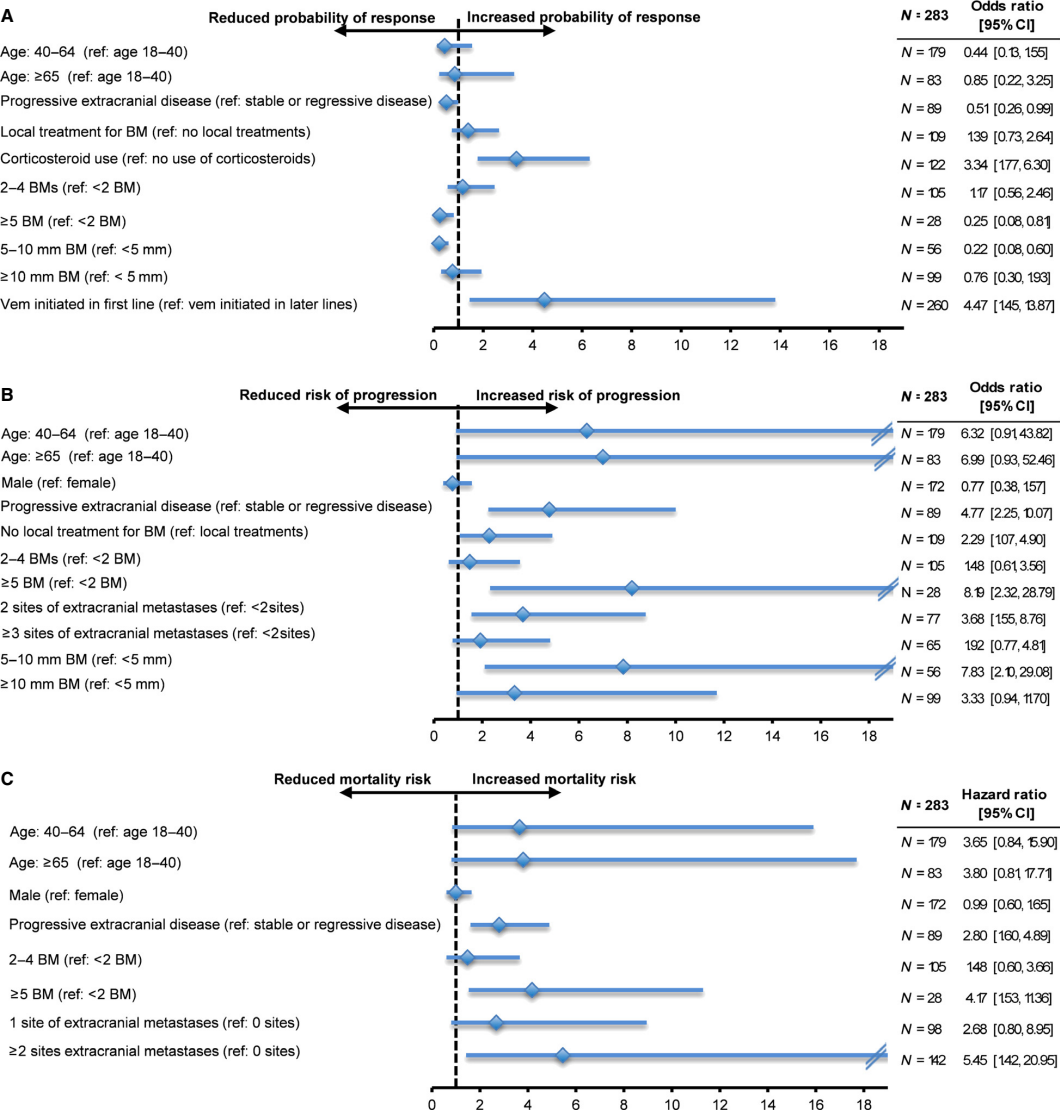
Factors associated with intracranial treatment response (A), intracranial disease progression (B), and mortality (C).

Patients receiving corticosteroids with vemurafenib (vs. no concomitant use) and patients receiving vemurafenib as a first-line systemic treatment (vs. later lines) following the diagnosis of brain metastases were associated with an increased likelihood of achieving an intracranial response by 3.34- and 4.47-fold, respectively (Fig.2A). Conversely, patients with ≥5 brain metastases (relative to one), larger intracranial tumor size (5–10 mm vs. <5 mm) and progressive extracranial disease at the time of vemurafenib initiation were less likely to achieve a treatment response by 75%, 78%, and 49%, respectively.

For intracranial disease progression while treated with vemurafenib, four factors associated with an increased risk included progressive extracranial metastasis at the time of vemurafenib initiation, presence of ≥5 brain metastases (relative to one), two extracranial metastatic sites (relative to one), and larger brain tumor size (5–10 mm relative to <5 mm) (Fig.2B). These factors increased the risk of disease progression by 4.77-, 8.19-, 3.68-, and 7.83-fold, respectively. Patients who did not receive local treatments prior to vemurafenib initiation were at 2.29-fold greater risk of intracranial disease progression.

Lastly, three clinical factors were associated with mortality risk: progressive extracranial metastatic status at time of vemurafenib initiation, presence of ≥5 brain metastases (relative to one), and ≥2 sites of extracranial metastases (Fig.2C). These factors increased the risk of mortality by 2.80-, 4.17-, and 5.45-fold, respectively.

## Discussion

Data from clinical trials suggest selective BRAF inhibitors have clinical activity in patients with BRAF V600-mutant MBM 17, 22, 23. Intracranial response rates range from 20% to 40% with median PFS of up to 4 months. More importantly, clinical activity was demonstrated in patients who failed local management of MBM with surgery or radiotherapy, a population where further treatment options are limited and prognosis is generally poor.

While randomized controlled trials have the highest level of causal inference, observational studies afford the opportunity to characterize outcomes in a broader population. Results from our study suggest improved outcomes associated with vemurafenib. A relatively high proportion of patients achieved intracranial (48.1%) and extracranial (45.6%) response. Survival rates estimated at 6 and 12 months following vemurafenib initiation were also relatively high; 85.6% at 6 months and 59.0% at 12 months. At the time of the data collection 53.0% of patients had discontinued vemurafenib. The main reasons reported for vemurafenib discontinuation, systemic disease progression and intracranial disease progression were consistent with the labeled indication which recommends continuing until disease progression or occurrence of unacceptable toxicity. One patient (0.7%) discontinued vemurafenib due to drug toxicity/intolerance. Although response and OS rates from this study were generally higher than results from previous clinical trials, comparisons with clinical trials should be approached with caution. Response and survival were based on physician assessment in a nonprotocol evaluation and the definitions utilized for CR/PR were less strict than RECIST or other objective criteria. The low proportion of patients who died during the observation period may also not be directly comparable to the rate found in clinical trials due to the short follow-up in our study. Nonetheless, our study suggests benefit of vemurafenib in MBM patients from the perspective of community and academic oncologists practicing in the real world.

This study sought to identify factors associated with better or worse outcomes in BRAF V600E-mutant MBM patients treated with vemurafenib. While there was no comparator arm to determine whether these factors are prognostic versus predictive, these variables may inform stratification in analyses of future trials or assist providers in patient care decisions. Our results showed that patients with more and larger brain metastases were less likely to achieve intracranial response. This is consistent with the slightly higher objective intracranial response rate seen in the phase II MBM study with dabrafenib compared to the phase II MBM study with vemurafenib, where the patient population treated with dabrafenib had fewer patients with >4BMs (21% vs. 33%) 17, 23. Past studies of MBM patients have also shown worse survival outcomes in patients with a large number of brain metastases, compared to only 1–3 brain metastases 6, 26, 27. However, these have been influenced by the ability to have localized intervention (surgery or SRS) in patients with fewer brain metastases versus WBRT in patients with many brain metastases. Unlike previous studies, we observed no association between elevated LDH or low-performance status and poor patient outcomes 6, 17, 27 However, this may be due to the relatively high proportion of patient with unknown LDH (25%) in our study, to the manner in which data were collected, or, to differences in the patient population.

This study is subject to common limitations inherent to retrospective studies using patient chart data. First, medical chart data are not collected for comparative research purposes. Variations in data collection, physician reporting, and loss to follow-up may exist. Second, data were available for a relatively short duration following vemurafenib initiation. This was due to vemurafenib’s recent approval and the need for sufficient sample size in a rare disease population. The study may also have been subject to self-selection bias, as participating physicians may differ in treatment patterns from the overall population of providers treating MBM patients. Third, our analysis did not account for variations in patient behavior. Adherence to treatment and clinic visits, for example, could impact outcomes related to response, disease progression, and survival. Lastly, our study did not conduct comparative analyses to assess the benefits of vemurafenib versus other treatments in MBM patients. Further studies are warranted to compare outcomes with other existing modes of treatment.

Despite these limitations, our study provides clinically meaningful insight into treatment outcomes for BRAF V600E-mutant MBM patients. These results can help clinical decision making in a rapidly evolving therapeutic landscape. This is particularly relevant with the shifting practice of combining BRAF inhibitors with selective MEK inhibitors in BRAF V600-mutant melanoma patients. Resistance to single-agent BRAF inhibitors occurs in most patients, which is frequently associated with reactivation of the MAPK pathway 19. Combination therapy with BRAF and MEK inhibitors has been shown to improve response rates and prolong survival when compared to single-agent BRAF inhibitors 23, 28–30. Increased objective response rates and PFS for extracranial disease have been reported for dabrafenib plus trametinib, as well as the combinations of vemurafenib plus cobimetinib and LGX818 plus MEK162 28, 29, 31–33. The availability of new BRAF and immune therapies compels questions surrounding optimal treatment sequencing for patients with MBM.

## Conclusion

The results from this retrospective chart review provide evidence of effectiveness of vemurafenib in BRAF V600E-mutant MBM patients. Nearly half of patients achieved both intracranial response and extracranial responses to vemurafenib. Factors associated with treatment outcomes were consistent with previous literature. Prospective studies are warranted to further characterize these clinical variables as predictive biomarkers for selecting MBM patients who benefit most from vemurafenib as a single agent or in combination with a MEK inhibitor.

## Appendix 1 Overall Survival Rates

**Table:**

	90-day	180-day	360-day
Original data collection			
All patients, *n* (%)	215 (76.0)	142 (50.2)	41 (14.5)
OS rate (95% CI)	94.2 (90.5, 96.5)	85.6 (80.1, 89.8)	59.0 (49.5, 67.2)
Patients with local treatments, *n* (%)	85 (78.0)	47 (43.1)	10 (9.2)
OS rate (95% CI)	94.8 (88.0, 97.8)	81.8 (70.9, 88.9)	61.4 (45.9, 73.6)
Patient without local treatments, *n* (%)	130 (74.7)	95 (54.6)	31 (17.8)
OS rate (95% CI)	93.8 (88.8, 96.6)	87.9 (81.0, 92.3)	59.4 (47.7, 69.3)
Physician recontact			
All patients, *n* (%)	250 (88.3)	181 (60.4)	82 (29.0)
OS rate (95% CI)	94.2 (90.6, 96.4)	84.1 (78.9, 88.1)	59.9 (52.4, 66.5)
Patients with local treatments, *n* (%)	98 (89.9)	70 (64.2)	33 (30.3)
OS rate (95% CI)	94.3 (87.8, 97.4)	79.8 (70.4, 86.5)	59.7 (48.5, 69.3)
Patient without local treatments, *n* (%)	152 (87.4)	111 (63.8)	49 (28.2)
OS rate (95% CI)	94.1 (89.3, 96.8)	87.0 (80.5, 91.4)	60.0 (49.9, 68.7)
Patients with a minimum follow-up duration			
30-day minimum, *N* = 262			
All patients, *n* (%)	215 (82.1)	142 (54.2)	41 (15.6)
OS rate (95% CI)	94.1 (90.4, 96.4)	85.6 (80.0, 89.7)	58.9 (49.5, 67.2)
60-day minimum, *N* = 247			
All patients, *n* (%)	215 (87.0)	142 (57.5)	41 (16.6)
OS rate (95% CI)	93.9 (90.0, 96.3)	85.4 (79.7, 89.5)	58.8 (49.4, 67.1)
90-day minimum, *N* = 230			
All patients, *n* (%)	215 (93.5)	142 (61.7)	41 (17.8)
OS rate (95% CI)	93.5 (89.4, 96.0)	85.0 (79.3, 89.3)	58.5 (49.1, 66.8)

OS, overall survival; CI, confidence interval.
